# The chain mediating effect of spiritual well‐being and anticipatory grief between benefit finding and meaning in life of patients with advanced lung cancer: Empirical research quantitative

**DOI:** 10.1002/nop2.2179

**Published:** 2024-06-28

**Authors:** Qingyue Luo, Fanglin Liu, Zhaoyu Jiang, Lan Zhang

**Affiliations:** ^1^ The First Affiliated Hospital of Jinzhou Medical University Jinzhou Liaoning Province China; ^2^ The Second Affiliated Hospital of Dalian Medical University Dalian Liaoning Province China

**Keywords:** advanced lung cancer, anticipatory grief, benefit finding, chain mediating effect, meaning in life, spiritual well‐being

## Abstract

**Aim:**

This study aimed to explore the chain mediating effect of spiritual well‐being and anticipatory grief between benefit finding and meaning in life of patients with advanced lung cancer.

**Design:**

This was a cross‐sectional study.

**Methods:**

The research included 400 patients with advanced lung cancer who attended REDACTE from December 2022 to August 2023 as the research subjects. Data were collected using a questionnaire including socio‐demographic and clinical characteristics, the Functional Assessment of Chronic Illness Therapy‐Spiritual Well‐Being scale (FACIT‐Sp‐12), the Benefit Finding Scale (BFS), the Preparatory Grief in Advanced Cancer Patients Scale (PGAC), and the Meaning of Life Questionnaire (MLQ). The structural equation model (SEM) was used to analyse the relationship between benefit finding, spiritual well‐being, anticipatory grief and meaning in life.

**Results:**

There was a significant correlation between benefit finding, spiritual well‐being, anticipatory grief, and meaning in life. Benefit finding could have a direct positive impact on meaning in life of patients with advanced lung cancer, but it could also indirectly affect meaning in life of patients with advanced lung cancer through three pathways: the mediating effect of spiritual well‐being, the mediating effect of anticipatory grief and the chain mediating effect of spiritual well‐being and anticipatory grief. Nursing staff should develop an integrated program of interventions to enhance the meaning in life of patients with advanced lung cancer.

## 1 INTRODUCTION

Primary bronchogenic carcinoma, commonly known as lung cancer, is the most prevalent malignant tumour in China, ranking first in both incidence and mortality rates (Wu et al., 2021). The onset of lung cancer is often insidious, with the majority of patients already in advanced stages at diagnosis. Patients with advanced lung cancer not only endure the physical pain caused by the tumour itself but also experience various physiological discomforts induced by different treatment modalities (Ester et al., 2021). Additionally, the exorbitant costs associated with treatment further exacerbate the economic burden on patients. Consequently, the majority of patients face significant pressures from various aspects including social, psychological and physiological, leading to a diminished sense of meaning in life among those with advanced lung cancer (Melosky et al., 2021). In the face of extreme stress, the protective role of meaning in life on psychological well‐being has been evidenced. When confronting life challenges and adversity, individuals endeavour to discern the essence of life, which aids in alleviating physical and psychological symptoms (Melosky et al., 2021). The exploration of meaning in life can provide patients with profound spiritual comfort and psychological support. The meaning in life can promote the healthy operation of the immune system, alleviate inflammatory responses (Liu, Wang, & Chiou, 2023), and enhance the body's recovery capabilities (Liu et al., 2021), thereby reducing the incidence of complications. Thus, investigating the factors influencing the meaning in life of patients with advanced lung cancer becomes particularly important. Benefit finding is a psychologically beneficial phenomenon for individual growth and development, enabling individuals to derive positive gains and experiences from negative experiences (Liu, Thong, et al., 2023). It is a significant factor influencing the sense of meaning in life. Spiritual well‐being, as an important psychological resource, plays a crucial role in assisting patients in coping with the challenges of illness and finding meaning in life (Soriano & Calong Calong, 2021). Additionally, anticipatory grief, as a psychological preparedness response to facing death and loss, has been shown to weaken patients' perception of meaning in life (Bermejo Gómez et al., 2023). These factors are interrelated. Although benefit finding, spiritual well‐being, and anticipatory grief play important roles in the lives of patients with advanced lung cancer, their specific mechanisms in the process of meaning in the life of discovery have not been fully studied. This study aims to explore the mechanisms of action of these four variables, with the potential to provide new theoretical support and practical guidance for psychological health interventions for patients with advanced lung cancer, helping them better cope with the disease and discover the meaning and value of life.

## 2 BACKGROUND

Lung cancer is a malignant tumour of the lung originating from bronchial mucosal epithelial cells and is the malignancy with the highest incidence and mortality rate worldwide. According to statistics, there will be approximately 2.2 million confirmed cases of lung cancer and up to 1.8 million deaths worldwide by 2022 (Siegel et al., 2022). Most lung cancer patients are asymptomatic in the early stages. They are already advanced at the time of initial diagnosis, and despite advances in treatment, the long‐term prognosis remains poor, with a low 5‐year survival rate (Bircan et al., 2020). Patients with advanced lung cancer have to deal with the health woes that come along with the disease, such as organ metastasis, pain due to long‐term radiotherapy, difficulty in breathing, and so on (Pribadi et al., 2023). These can lead to the deterioration of the patient's psychological condition, generating a high degree of negative emotions and reducing the patient's sense of meaning in life (Ester et al., 2021; Villalobos et al., 2019).

Patients with advanced lung cancer face complex challenges in both treatment and daily life. Against this backdrop, paying attention to patients' psychological and spiritual aspects, especially exploring the meaning of life, becomes crucial. Park categorized the meaning in life into global meaning and situational meaning. Global meaning refers to a general orienting system encompassing individuals' core assumptions about life's goals and hierarchical ordering. It involves subjective perceptions of coherence, purpose and significance. This implies that, when facing life and death moments, patients may reevaluate their beliefs, objectives and subjective feelings, searching for the overall meaning in life (Sherman et al., 2022). Elements such as faith, family and interpersonal relationships may play crucial roles within this global meaning. These become essential for patients to comprehend life goals and coherence (Littooij et al., 2016). Situational meaning refers to the meaning derived from specific situational encounters (Sherman et al., 2022). For patients with advanced lung cancer, assessing the impact of the disease on life is a crucial aspect. The dynamic changes in treatment and the progression of the illness may trigger a process of assigning new meaning to traumatic events. Patients need to find a balance between global and situational meaning, constructing meaning to cope with traumatic events (Nakayasu, 2019). Furthermore, Steger defines meaning in life as an individual's perception of their own existence and the meaning of that existence, including the awareness and perception of significant things or others (Conner et al., 2022). In patients with advanced lung cancer, exploration at this level may be more complex. Patients need to perceive changes in their existence and recognize the importance of family, friends and healthcare providers in their lives. This recognition may profoundly influence the meaning in the lives of patients, guiding them to find meaning in moving forward in the shadow of the disease (Jadidi & Ameri, 2022). Overall, the meaning in life of patients with advanced lung cancer involves the intertwining of global meaning and situational meaning, along with a profound perception of their existence. Patients may reevaluate beliefs, objectives and significant relationships during this process. Simultaneously, they may seek deeper meaning in life within the context of the disease and treatment. This comprehensive construction of meaning in life helps patients better cope with the irreversibility of life.

Benefit finding pertains to the positive outcomes individuals obtain from traumatic events, such as illness. These gains manifest in four dimensions: personal, social, psychological and spiritual growth (Lassmann et al., 2021). Experiencing benefit findings can lead patients to develop positive cognitions when facing stressful events, which is conducive to enhancing their self‐worth. The realization of self‐worth enables individuals to attain more positive emotional experiences and possess intrinsic strength, which is crucial for improving a sense of meaning in life (Carreno et al., 2023). A specific connection between benefit finding and meaning in life is evident. Patients with advanced lung cancer often undergo a series of emotional, psychological and physiological changes when facing the challenges of a terminal illness. In this process, some patients demonstrate profound reflections on life, re‐examining and redefining their meaning through the lens of benefit finding (Temel et al., 2022). This benefit finding is not a simple, singular event but rather a complex psychological process involving multiple facets of experiences and perceptions. Initially, patients in the terminal stage often reassess past life choices and values through benefit finding. This reassessment may involve reflections on personal achievements, relationships, and the meaning of life. When facing the fragility and transience of life, patients gradually recognize some overlooked crucial elements, thereby imbuing new meaning into their lives. This process is a cognitive shift and an internal exploration, assisting patients in finding a more profound sense of existence (Zhou et al., 2023). Furthermore, benefit finding appears closely linked to improving and deepening interpersonal relationships. In the terminal stage, patients with higher levels of benefit finding tend to prioritize their families, friends and intimate connections. By sharing gratitude and expressing love with their loved ones, they establish stronger emotional bonds during this challenging time. This deepening of interpersonal relationships provides patients with more support and injects richer emotional experiences into their lives, adding precious meaning to their existence. (Kaufhold et al., 2023). Simultaneously, benefit finding enables patients to seek beauty and experience happiness in the present moment through a more nuanced perceptual approach. This present‐focused attention allows patients to distance themselves from the uncertainties and pain of the future, concentrating on the positive aspects of life, ultimately enhancing the quality of life (Ma et al., 2023). It can be observed that benefit findings significantly influence meaning in life, a viewpoint supported by Manne's investigation into cervical cancer patients (Manne et al., 2018). Therefore, we propose hypothesis 1 that benefit finding positively impacts meaning in life.

Spiritual well‐being refers to an individual's affirmation of self, others, and the value of the environment. It involves understanding the meaning of life, fostering harmonious interactions with others, society, and the environment and the capacity to adapt to adversity (Juškauskienė et al., 2023). In recent years, research on spiritual well‐being has been increasing, with the World Health Organization listing spiritual well‐being as an important protective factor for health (Ghourchian et al., 2023). Spiritual well‐being involves not only experiences of religious beliefs but also the pursuit of life purpose, meaning, and a sense of connection. By deeply understanding the spiritual well‐being status of patients with advanced lung cancer, we can comprehensively grasp their reflections and experiences of life (Li et al., 2021). Firstly, spiritual well‐being often manifests in patients with advanced lung cancer as a quest for inner strength and meaning. Facing the threat of life, patients may seek spiritual support, attempting to find inner peace and tranquillity through contemplation, meditation, or religious rituals (Shin et al., 2018). This process of seeking spirituality helps patients establish a deeper understanding of life, transcending physiological pain and experiencing a profound sense of existence. Secondly, spiritual well‐being also involves a sense of connection with others, nature and the universe. Patients may experience a transcendent connection beyond the individual, feeling resonance with others and nature during this stage (Abdalrahim et al., 2023). This sense of connection helps patients transcend the loneliness of end‐of‐life, making them feel part of a larger whole in life. By sharing this connection with others, patients can find support and understanding in intimate relationships, infusing life with deeper meaning. Additionally, spiritual well‐being also involves reflection on life's purpose and values (Xia et al., 2023). Patients may re‐evaluate their life goals during the late stages of lung cancer, pondering the meaning of their existence in this world. This pursuit of deeper life purpose enables patients to recognize more clearly the tiny yet precious position of their individual lives in the entire universe, thus imbuing life with deeper meaning. From this, we can see that spiritual well‐being can enhance the sense of meaning in life, consistent with Sleight and Aydın's studies (Aydın et al., 2020; Sleight et al., 2021). Therefore, we propose hypothesis 2 that spiritual well‐being positively impacts meaning in life.

Anticipatory grief encompasses both emotional responses and behaviours arising from individuals' perception of the potential loss of someone or something significant, which may alter their self‐concept (Coelho et al., 2018). Patients with advanced lung cancer often experience a sense of anticipatory grief as they approach the final stages of life, which may in some cases, affect their understanding of the meaning in life. Anticipatory grief can lead to poor compliance with cancer treatment, prolong the recovery time from the illness, and further deteriorate the overall health status of patients. These feelings can break down the psychological defences of advanced cancer patients, leading to feelings of guilt, worthlessness, despair, and helplessness (Bilić et al., 2022). Anticipatory grief is not simply an emotional fluctuation but more like a shadow creeping silently over the heart, causing some changes in the patient's deep‐seated understanding of life's meaning. Patients may perceive the instability and transience of life in the context of anticipatory grief. This is not an immediate cause‐and‐effect result but rather a heightened sensitivity to the essence of life within the atmosphere of sadness, making life's meaning seem more fragile and elusive (Barber, 2022). Additionally, anticipatory grief may trigger profound reflections on past life experiences for patients. During this stage, patients' retrospection on life's past appears with heavier tones, highlighting more regrets and disappointments rather than moments of beauty and success, thereby diminishing the sense of meaning derived from past life experiences (Rogalla, 2020). Li's study revealed a significant negative correlation between anticipatory grief and meaning in life (Li et al., 2022). Therefore, we propose hypothesis 3 that anticipatory grief negatively impacts on meaning in life.

Additionally, these factors are interconnected. The benefit findings in patients with advanced lung cancer can have a positive impact on their spiritual well‐being. Benefit finding assists patients in cultivating inner peace and acceptance, enabling them to better cope with the realities of the disease and approach their life journey with a positive mindset. This inner peace and acceptance help patients establish more solid internal support, leading to higher levels of satisfaction and tranquillity at a spiritual level (Wen et al., 2022). Carrico's research on HIV‐positive individuals found that benefit findings can influence spiritual well‐being to a greater extent, enabling individuals to more effectively manage the cumulative effects of HIV infection (Carrico et al., 2006). Therefore, we propose hypothesis 4 that benefit finding positively impact spiritual well‐being. Combining benefit finding and spiritual well‐being's impact on meaning in life, we propose hypothesis 5 that spiritual well‐being mediates the relationship between benefit finding and meaning in life. Researchers have found a significant negative correlation between benefit finding and anticipatory grief (Prikken et al., 2022). Furthermore, individuals with high levels of benefit finding reduce the negative impact of stressors through cognitive adaptation, finding value in themselves amid adversity for self‐improvement (de Vries et al., 2019). This self‐improvement enables patients to achieve a more favourable balance before and after illness, regain control over their lives, overcome feelings of helplessness, and reduce levels of anticipatory grief. Therefore, we propose hypothesis 6 which benefits finding negatively impacts anticipatory grief. Combining the influence of benefit finding on meaning in life and the connection between anticipatory grief and benefit finding, we propose hypothesis 7 that anticipatory grief mediates the relationship between benefit finding and meaning in life. Mendes' research has found that spiritual well‐being provides patients with a pathway to seek inner peace and tranquillity. When facing the challenges of treatment and discomfort associated with late‐stage cancer, patients may experience various negative emotions, including anxiety and despair. Through spiritual practices, patients can find an internal support system, experiencing a calmness that transcends illness and pain, thereby mitigating the fluctuations of sorrowful emotions (Mendes et al., 2023). Additionally, spiritual well‐being can assist patients in developing a more serene attitude towards death. Faced with the irreversible process of advanced lung cancer, patients may experience fear and anxiety about death. By cultivating spiritual well‐being, patients have the opportunity to explore a profound understanding of life's end, thereby better accepting death as a part of the life cycle, reducing anticipatory grief about the unknown (Ata & Kılıç, 2022). Therefore, we propose hypothesis 8 that spiritual well‐being negatively impacts anticipatory grief. Building upon the cumulative findings, we propose hypothesis 9, that spiritual well‐being and anticipatory grief play a chain mediating role between benefit finding and meaning in life.

**FIGURE 1:**
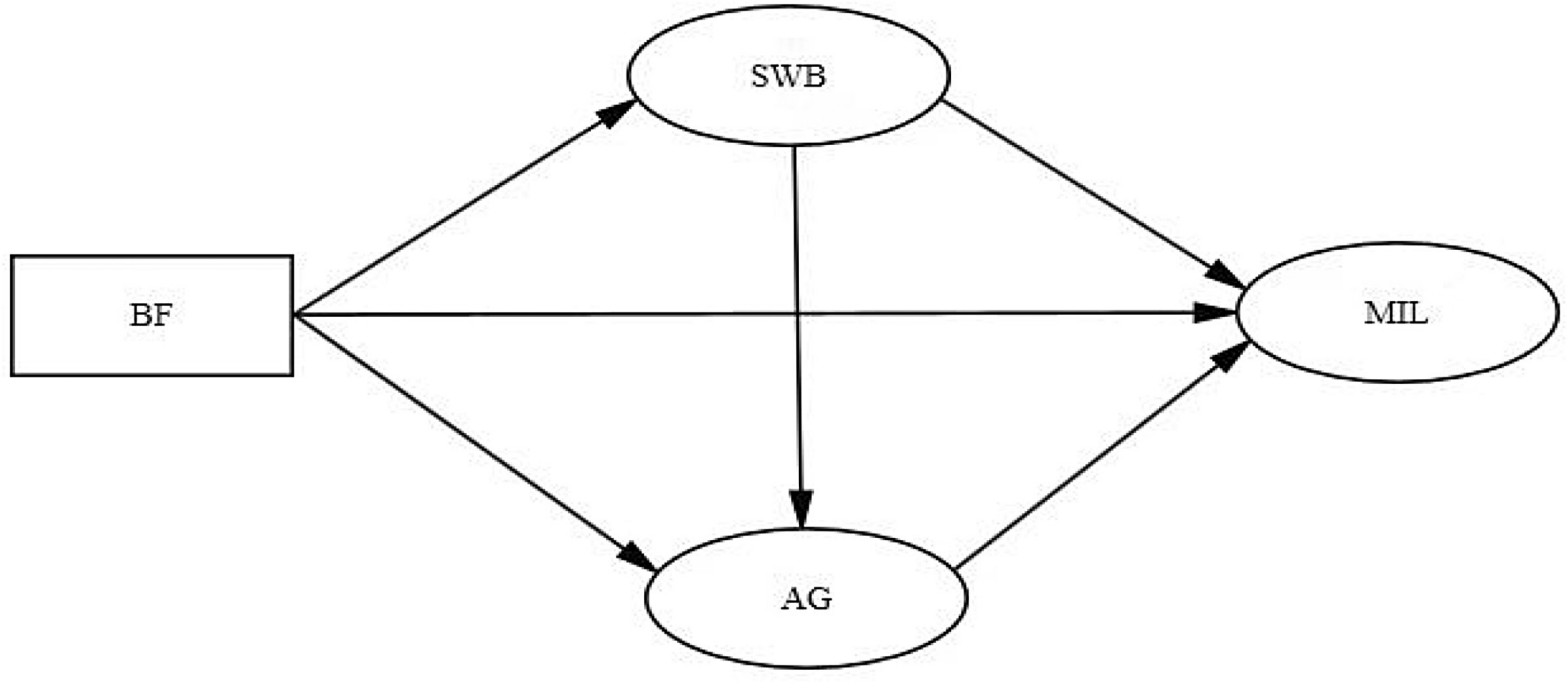
Conceptual model. AG, anticipatory grief; BF, Benefit finding; MIL, Meaning in life; SWB, Spiritual well‐being.

Based on previous studies, we propose the above hypotheses and a theoretical model (Figure 1). This study explored the mechanism of action between these variables and aimed to provide a theoretical basis for improving the meaning in life of patients with advanced lung cancer.

### 2.1 Theoretical basis

The stress and coping theory proposed by Lazarus (Lazarus, 1993), suggested that individuals when faced with a stressful event, first engaged in cognitive evaluation. Individuals perceived whether the situation had an impact on them, and then responded to the stressful event to solve the problem (e.g., to improve life meaning) based on available information and experience of illness. The diagnosis of advanced lung cancer served as the beginning point for this study's examination of psychological stressors, benefit finding as a cognitive evaluation, anticipatory grief, and spiritual well‐being as the manifestation of coping styles, meaning in life as a response result.

## 3 METHODS

### 3.1 Study design and participants

This was a cross‐sectional study in which patients with advanced lung cancer were recruited from December 2022 to August 2023 at the inpatient department of REDACTED using a convenience sampling method. Inclusion criteria: (1) Pathological diagnosis of lung cancer, (2) Age ≥ 18 years old, (3) The disease stage was III or IV, (4) Ability to read the text to complete the questionnaire, (5) Voluntary participation in this study. Exclusion criteria: unaware of the disease, cognitive impairment or delirium, and those with other significant disorders in combination. According to Kendall's guidelines, the sample size should be at least 5–10 times the number of observed independent variables (Rossi et al., 2021). In this study, the questionnaire included a total of 8 variables covering general demographic and disease‐related data, 1 dimension for the Benefit Finding Scale, 3 dimensions for the Spiritual well‐being Scale, 5 dimensions for the Anticipatory Grief Scale, and 2 dimensions for the Meaning in Life Scale. Therefore, the total number of independent variables included in this study is 19, at least requiring a sample size of 95–190 cases. The final sample size was 400 cases.

### 3.2 Data collection

Upon obtaining consent from the department head and nursing supervisor, investigators first screened electronic medical records at the nurse stations of the relevant departments. Then, they proceeded to patient wards that met the inclusion criteria and explained the purpose of the survey and the questionnaire‐filling method to the patients. After the patients fully understood and signed the informed consent form, the investigators distributed the questionnaires on the spot. Patients filled out the questionnaires according to their actual situation. The investigators recorded their responses based on the patients' oral instructions for those unable to fill out the questionnaire due to uncontrollable factors such as intravenous infusion. While filling out the questionnaire, investigators could not suggest answers to the patients. It took each person approximately 15–20 min to complete the questionnaire.

### 3.3 Ethical approval

This study has been approved by the Ethics Committee of The First Affiliated Hospital of Jinzhou Medical University, with approval number JZMULL2023050. All participants signed written informed consent prior to participating in the study.

### 3.4 Assessments

Socio‐demographic and clinical characteristics included age, gender, education level, spouse status, religious belief status, monthly income, disease stage and disease duration.

Spiritual well‐being was assessed using the Functional Assessment of Chronic Illness Therapy‐Spiritual Well‐Being scale (FACIT‐Sp‐12). The scale was developed by Brady (Brady et al., 1999) and has been validated in the Chinese population with a validated Cronbach's *α* of 0.831. The scale has three dimensions, meaning, Peace, and Faith, with 12 items. The scale adopts the Likert‐5‐level scoring method, with each item scoring from 0 (not at all) to 4 (very much). Among them, items 4 and 8 are achieved in reverse, with a total score of 0–48 points. The higher the scale score, the better the patient's spiritual well‐being. In this study, the FACIT‐Sp‐12 showed good consistency (Cronbach's *α* = 0.817).

Benefit finding was assessed using the benefit finding scale (BFS) initially developed by Antoni (Antoni et al., 2001). The scale has one dimension and has been validated in the Chinese population with Cronbach's *α* of 0.91. Chinese scholars have revised it to consider the current situation in China. The scale adopts the Likert‐4‐level scoring method, including 19 items, with each item scored from 1 (not at all) to 4 (very much) and a total range of 19–76, with higher scale scores indicating more patient benefit findings. In this study, the BFS showed good consistency (Cronbach's *α* = 0.758).

Meaning in life was assessed using the Meaning in Life Questionnaire (MLQ). The scale was first developed by Steger, including two dimensions (presence and seeking) and ten items (King & Hicks, 2021). The scale has been validated in the Chinese population with Cronbach's *α* of 0.77. Chinese scholars have revised it to consider the current situation in China by removing one item from the original scale. The scale adopts the Likert‐7‐level scoring method, with each item scored from 1 (not at all) to 7(very fully). Item 2 is reverse scoring, and all other items are positive, with a total score range of 9–63, with higher scores representing a stronger sense of meaning in an individual's life. In this study, the MLQ showed good consistency (Cronbach's *α* = 0.703).

Anticipatory grief was initially assessed using the Preparatory Grief in Advanced Cancer Patients Scale (PGAC), developed by Mystakidou with 31 items (Mystakidou et al., 2005). The scale has been validated in the Chinese population, and the validated scale of Cronbach's *α* is 0.919. Chinese scholars have revised it in the context of the current situation in China by removing five items from the original scale. The fixed scale contains 26 items with five dimensions: grief and anger, attitude toward death, attitude toward somatic symptoms, religious comfort, and perceived social support. The scale adopts the Likert‐4‐level scoring method, with each item scored from 0 (disagreement) and 3 (agreement), with a total score range of 0–78. The higher the score on the scale, the higher degree of anticipatory grief. In this study, the PGAC showed good consistency (Cronbach's *α* = 0.945).

### 3.5 Data analysis

SPSS 24.0 was used to analyse the data, and statistical significance was set at *p* < 0.05. Count data were described by frequency and percentage, and measurement data by mean‐standard deviation (Mean ± SD). Independent samples t‐test and One‐way analysis of variance (ANOVA) were used to compare differences in socio‐demographic and clinical characteristics on dependent variable scores. Pearson analysis of correlations between variables was used, and AMOS 24.0 was used to construct structural equations (SEM) to test the hypotheses outlined in the conceptual model (Figure 1). We tested a sample of 5000 using a bootstrap method which computed the bias‐corrected 95% confidence interval (CI), and the results were considered significant if the 95% CI excludes zero. The Chi‐square degree of freedom ratio (χ^2^/*df*) < 3, Standardized root mean square residual (SRMR) <0.050, Root mean square error of approximation (RMSEA) <0.08, Goodness of fit index (GFI) >0.900, Adjusted goodness of fit index (AGFI) >0.900, Comparative fit index (CFI) >0.900, Tucker–Lewis index (TLI) >0.900, Incremental fit index (IFI) >0.900 were used to confirm the model fit.

## 4 RESULTS

### 4.1 Participant characteristics

This study surveyed a total of 400 patients. A majority of them were over the age of 60 (238,59.5%). Among the participants, most were male (263, 65.75%). The impact of education level on the sense of meaning in life was the most significant. Detailed demographic information can be found in Table 1.

**TABLE 1:** Socio‐demographic and clinical characteristics (*N* = 400).

Variables	*N* (%)	Meaning in life	t/F	*p*
Age				
<45 years old	22 (5.50)	34.50 ± 5.078	3.996	0.019*
45–60 years old	140 (35.00)	36.74 ± 7.035		
>60 years old	238 (59.5)	38.31 ± 7.719		
Gender				
Male	263 (65.75)	37.60 ± 7.511	0.194	0.846
Female	137 (34.25)	37.45 ± 7.272		
Education level			7.924	0.000***
Primary school and below	124 (31.00)	35.52 ± 7.430		
Middle school	136 (34.00)	37.31 ± 7.297		
High school/technical secondary school	86 (21.5)	38.80 ± 7.377		
Junior college	54 (13.5)	40.85 ± 6.332		
Spouse status			2.252	0.029*
Have a spouse	359 (89.75)	37.90 ± 7.118		
No spouse	41 (10.25)	34.54 ± 9.247		
Religious belief status			−2.275	0.028*
Have religious belief	41 (10.25)	40.51 ± 8.961		
Have no religion belief	359 (89.75)	37.21 ± 7.162		
Monthly income			6.405	0.002**
<3000 Yuan	171 (42.75)	36.06 ± 8.508		
3000–5000 Yuan	154 (38.50)	38.46 ± 6.220		
>5000 Yuan	75 (18.75)	39.09 ± 6.433		
Disease stage			6.329	0.012*
III	188 (47.00)	38.54 ± 7.290		
IV	212 (53.00)	36.68 ± 7.445		
Disease duration			4.649	0.010*
<4 months	75 (18.75)	39.83 ± 8.811		
4–8 months	163 (40.75)	37.31 ± 7.032		
>8 months	162 (40.5)	36.75 ± 6.923		

* Significant at *p* < 0.05.

** Significant at *p* < 0.01.

*** Significant at *p* < 0.001.

### 4.2 Description of spiritual well‐being, benefit finding, anticipatory grief and meaning in life scores

The scores for each variable are shown in Table 2. The mean scores for benefit finding, spiritual well‐being, anticipatory grief and meaning in life were 55.83 ± 4.150, 29.09 ± 6.952, 48.35 ± 13.733, 37.55 ± 7.421. The dimension scores were 8.96 ± 2.804, 9.83 ± 2.718, 10.30 ± 3.355, 22.15 ± 7.169, 7.54 ± 2.458, 7.18 ± 2.831, 5.86 ± 2.201, 5.31 ± 2.237, 21.68 ± 4.889, 15.87 ± 3.952.

**TABLE 2:** Spiritual well‐being, benefit finding, anticipatory grief and meaning in life scores (Mean ± SD*, N* = 400).

Contents	Range	Mean ± SD
Benefit finding	19–76	55.83 ± 4.150
Spiritual well‐being	0–48	29.09 ± 6.952
Peace	0–16	8.96 ± 2.804
Meaning	0–16	9.83 ± 2.718
Faith	0–16	10.30 ± 3.355
Anticipatory grief	0–78	48.35 ± 13.733
Grief and indignation	0–36	22.15 ± 7.169
Death attitude	0–12	7.54 ± 2.458
Somatic symptoms	0–12	7.18 ± 2.831
Religious comfort	0–9	5.86 ± 2.201
Perceived social support	0–9	5.31 ± 2.237
Meaning in life	9–63	37.55 ± 7.421
Sense of presence	1–35	21.68 ± 4.889
Sense of seeking	1–28	15.87 ± 3.952

### 4.3 Correlation analysis

The correlations between benefit finding, spiritual well‐being, anticipatory grief and meaning in life are shown in Table 3. Benefit finding was positively associated with spiritual well‐being (*r* = 0.439, *p* < 0.01) and meaning in life (*r* = 0.359, *p* < 0.01). In contrast, benefit finding was negatively related to anticipatory grief (*r* = −0.335, *p* < 0.01). Spiritual well‐being was negatively associated with anticipatory grief (*r* = −0.246, *p* < 0.01) and positively associated with meaning in life (*r* = 0.303, *p* < 0.01). Anticipatory grief was negatively associated with meaning in life (*r* = −0.290, *p* < 0.01).

**TABLE 3:** Correlation between spiritual well‐being, benefit finding, meaning in life and anticipatory grief (*N* = 400).

Variable	BF	SWB	AG	MIL
BF	1			
SWB	0.439**	1		
AG	−0.335**	−0.246**	1	
MIL	0.359**	0.303**	−0.290**	1

Abbreviations: AG, anticipatory grief; BF, Benefit finding; MIL, Meaning in life; SWB, Spiritual well‐being.

** Significant at *p* < 0.01.

### 4.4 Structural equation model

**FIGURE 2:**
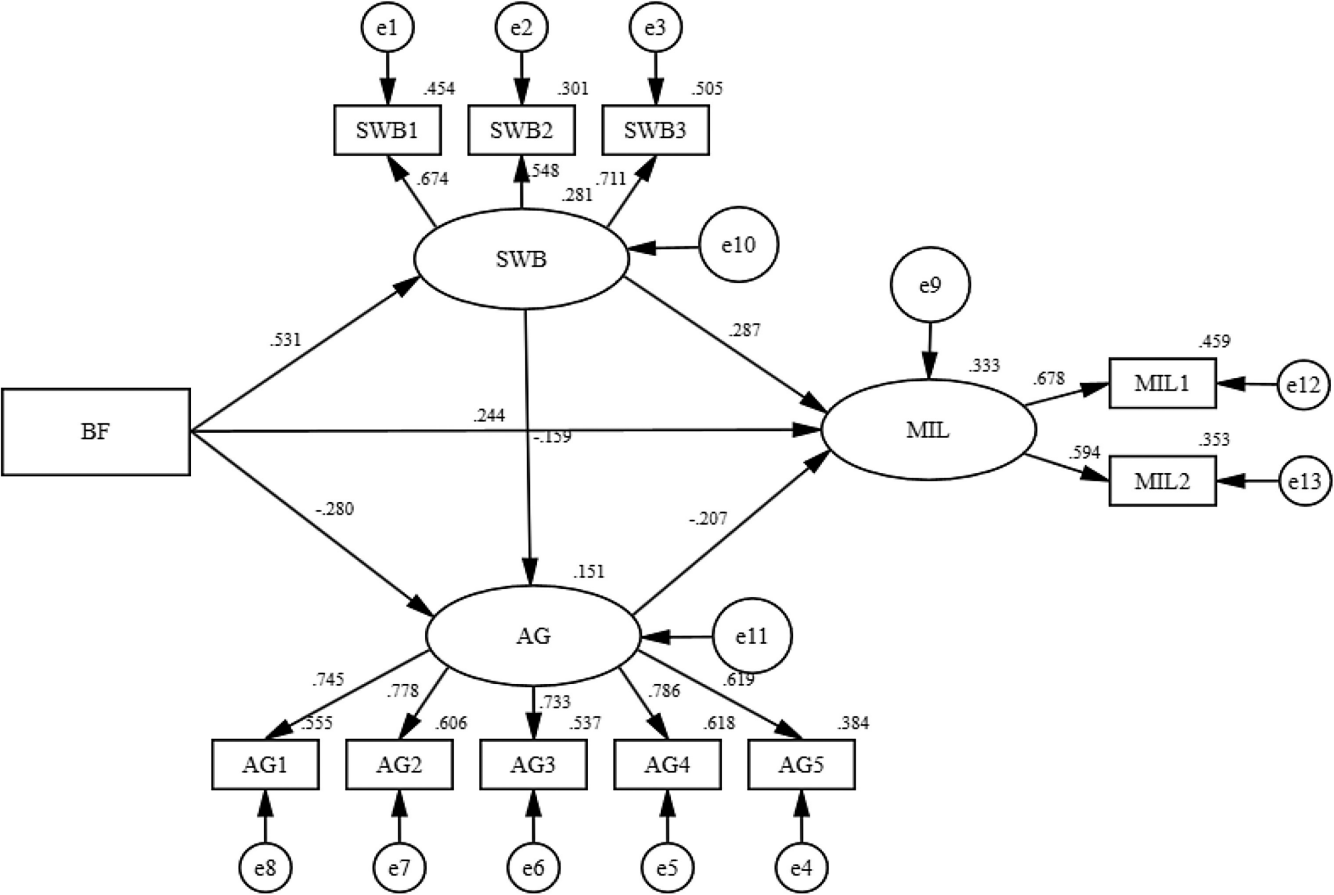
Standardized model.

The model was constructed with spiritual well‐being and anticipatory grief as the mediator. In this model, benefit finding was set as the independent variable, meaning in life was set as the dependent variable, and Figure 2 shows the results of SEM used to test the hypothesis of this study. The direct, indirect, and total effects are shown in Table 4. Benefit finding (*β* = 0.195, SE = 0.075, 95% CI [0.051, 0.347]) and spiritual well‐being (*β* = 0.504, SE = 0.189, 95% CI [0.194, 0.940]) had a positive effect on meaning in life, so hypotheses 1 and 2 were supported. Anticipatory grief had a negative effect on meaning in life (*β* = −0.496, SE = 0.159, 95% CI [−0.798, ‒0.187]), so hypothesis 3 was supported. Benefit finding had a positive effect on spiritual well‐being (*β* = 0.242, SE = 0.040, 95% CI [0.168, 0.325]), so hypothesis 4 was supported. Benefit finding had a negative effect on Anticipatory grief (*β* = −0.094, SE = 0.024, 95% CI [−0.139, −0.047]), so hypothesis 6 were supported. Spiritual well‐being had a negative effect on anticipatory grief (*β* = −0.117, SE = 0.051, 95% CI [−0.220, −0.019]), so hypothesis 8 was supported. Spiritual well‐being mediated the relationship between benefit finding and meaning in life (*β* = 0.122, SE = 0.048, 95% CI [0.047, 0.234]), so hypothesis 5 was supported. Anticipatory grief mediated the relationship between benefit finding and meaning in life (*β* = 0.046, SE = 0.019, 95% CI [0.016, 0.091]), so hypothesis 7 was supported. Anticipatory grief and spiritual well‐being played a chain mediating role between benefit finding and meaning in life (*β* = 0.014, SE = 0.008, 95% CI [0.003, 0.036]), and hypothesis 9 was supported. Therefore, all hypotheses in this study were supported. Indirect effect 1, Indirect effect 2, Indirect effect 3, Total indirect effect and Direct effect 6 accounted for 32.3%, 12.3% 3.7%,48.3% and 51.7% of the Total effect, respectively. Table 5 shows eight indicators (χ^2^/df = 1.749, SRMR = 0.0342, RMSEA = 0.043, GFI = 0.971, AGFI = 0.951, CFI = 0.977, TLI = 0.968, IFI = 0.978) confirming the goodness‐of‐fit of the model.

**TABLE 4:** Non‐standardized total, direct and indirect effects.

Path	Estimate	SE	Bias‐corrected Percentile 95% CI		Percentage of total effect (%)
			Lower	Upper	
Direct effect1:BF → SWB	0.242	0.040	0.168	0.325	
Direct effect2:BF → AG	−0.094	0.024	−0.139	−0.047	
Direct effect3:SWB → AG	−0.117	0.051	−0.220	−0.019	
Direct effect4:SWB → MIL	0.504	0.189	0.194	0.940	
Direct effect5:AG → MIL	−0.496	0.159	−0.798	−0.187	
Direct effect6:BF → MIL	0.195	0.075	0.051	0.347	51.7
Indirect effect1:BF → SWB → MIL	0.122	0.048	0.047	0.234	32.3
Indirect effect2:BF → AG → MIL	0.046	0.019	0.016	0.091	12.3
Indirecteffect3:BF → SWB → AG → MIL	0.014	0.008	0.003	0.036	3.7
Total indirect effect: BF → MIL	0.182	0.047	0.102	0.290	48.3
Total effect: BF → MIL	0.377	0.063	0.250	0.497	100.0

Abbreviations: AG, Anticipatory grief; BF, Benefit finding; MIL, Meaning in life; SWB, Spiritual well‐being.

**TABLE 5:** Results of the measurement model fitness test.

	χ^2^/*df*	SRMR	RMSEA	GFI	AGFI	CFI	TLI	IFI
Suggested value	<3	<0.050	<0.080	>0.900	>0.900	>0.900	>0.900	>0.900
Model value	1.749	0.0342	0.043	0.971	0.951	0.977	0.968	0.978

## 5 DISCUSSION

This study aimed to elucidate the interrelationship between spiritual well‐being, benefit finding, anticipatory grief and meaning in life in patients with advanced lung cancer. It is the first study to report the chain mediating role of anticipatory grief and spiritual well‐being between benefit finding and meaning in the life of patients with advanced lung cancer. The findings here may provide possible clues and directions for developing and implementing measures to enhance meaning in life in patients with advanced lung cancer.

### 5.1 Current status of spiritual well‐being, benefit finding, anticipatory grief and meaning in life for patients with advanced lung cancer

The mean score of the spiritual well‐being for patients with advanced lung cancer in the study was (29.09 ± 6.952), which was lower than the findings of Martoni for advanced cancer in Italy (Martoni et al., 2017), and was moderately low overall. There are sufficient resources for spiritual well‐being care in foreign countries, and more studies have been conducted on spiritual well‐being care. There is a lack of resources for spiritual well‐being care in China, and professionals are not competent enough to provide spiritual care. More importantly, Chinese healthcare professionals focus on the treatment status of patients with advanced cancer. They often neglect the care of the spiritual well‐being of patients with advanced cancer. At the same time, patients in China do not understand spiritual well‐being and have internal resistance to it, which results in relatively low spiritual well‐being scores (Cheng et al., 2021).

The mean score of benefit finding for patients with advanced lung cancer in the study was (55.83 ± 4.150), which was moderately high overall and higher than the results of Rong's survey on advanced cancer in China (Rong et al., 2022). The difference in findings may be related to the fact that there were more elderly patients over 60 years of age in this study. Elderly patients have richer social experiences, and therefore have more robust psychological tolerance. While they suffer physically and mentally, they can also feel the benefits of the disease and thus face the disease more openly.

The mean score of anticipatory grief for patients with advanced lung cancer in the study was (48.35 ± 13.733), which was moderately high overall and higher than the findings of Vergo for patients with advanced cancer in America (Vergo et al., 2017). Differences in cultural backgrounds and meanings in life screening and assessment tools in various national populations can cause differences in study results. On the contrary, China offers less palliative care for advanced cancer than foreign countries, which does not improve clinical symptoms, relieve patients' suffering and improve their quality of life to a greater extent (Hahne et al., 2022).

The mean score of meaning in life was (37.55 ± 7.421), which was low overall and lower than the findings of Gravier on the meaning in life of patients with advanced cancer (Gravier et al., 2020). Because the subjects of the current study were advanced lung cancer patients with more severe symptoms and rapid disease progression than other advanced cancer patients. There was a tremendous psychological fear of deterioration in the patients. Moreover, most of the advanced lung cancer patients in this study had low‐monthly incomes, and their attitude towards life started to become pessimistic due to the high cost of treatment.

The analysis of variance showed that age, education level, spousal status, religious belief status, monthly income, disease stage and disease duration were influential factors in the meaning in life of patients with advanced lung cancer. The older patients had a higher level of meaning in life, probably because older patients had more experience. They were more able to maintain a peaceful state of mind and more positive emotions after the disease (Chen, 2022). Patients with higher education levels demonstrated a heightened sense of meaning in life. This could be attributed to their increased access to social support networks and resources. Additionally, their enhanced capacity for understanding and analysing the disease encourages deeper introspection into life's meaning (Volkert et al., 2019). Patients who were married exhibited a higher sense of meaning in life, possibly due to the supportive role of their spouses. Spouses often served as attentive listeners and offered appropriate guidance to patients during times of emotional distress. Patients with religious beliefs had a higher meaning in life because they were more likely to find spiritual support and accept the truth about the disease to relieve their stress when facing pain (Gravier et al., 2020). The elevated level of meaning in life observed among patients with higher monthly incomes suggests that favourable economic circumstances and material resources contribute to a sense of security in life. This, in turn, may exert a positive influence on psychological well‐being (Aktürk & Erci, 2018). Patients in the advanced III stage of cancer exhibited a higher level of meaning in life. This could be attributed to the worsening symptoms accompanying disease progression, leading to increased mental stress and negative emotions regarding treatment patients with a shorter duration of illness demonstrated a higher level of meaning in life. This observation might be linked to the challenges faced by patients with longer illness durations, such as prolonged treatments, fluctuations in physical functioning, and psychological distress, all potentially contributing to a loss of self‐confidence (Krok et al., 2022).

Interestingly, the majority of participants in the study did not have religious beliefs, yet the belief dimension of spiritual well‐being was high. This is because although most Chinese people do not identify with any specific religion, due to the influence of traditional Chinese culture, especially Confucianism, Taoism and Buddhism, they still exhibit beliefs and behaviours similar to religious practices. This cultural background provides spiritual support akin to religious beliefs even for those who do not consider themselves religious (Hu et al., 2023). Religious beliefs enhance the meaning in life, a notion that is contradicted in studies on negative religious coping. A study suggests that religious beliefs can provide individuals with spiritual support and emotional comfort. Through faith, people can seek purpose and meaning in life, connecting their personal experiences to broader cosmic contexts, thus feeling more fulfilled and balanced (Womick et al., 2022). However, despite the positive psychological benefits that religious beliefs may bring to individuals, some studies also point out the potential negative impacts of negative religious coping strategies. In certain situations, excessive reliance on religious beliefs may lead to avoidance of real‐life issues and neglect of secular aspects of life. Additionally, some individuals may become involved in religious extremism or dogmatism, resulting in conflicts with other groups and social disconnection (Gravier et al., 2020; Yilmaz, 2019).

### 5.2 Benefit finding had a positive impact on meaning in life

Hypothesis 1 was supported as the finding indicated that benefit finding positively impacted meaning in life, consistent with previous research (Autio & Rissanen, 2018). Moreover, patients reported that benefit finding made capturing positive information about the treatment process more accessible, which was essential for perceiving the value of the existence of meaning in life (Chiba et al., 2021). Advanced lung cancer involves multiple physical, mental, spiritual and social traumas that could disrupt assumptions about the world and self and produce psychological distress. The core of benefit finding was for individuals to find positive or beneficial aspects of traumatic events, and would affect mental and physical health outcomes. It could trigger cognitive structural adjustments, enable patients to find valuable things for themselves in misfortune, and promote physical and psychological recovery (St Fleur et al., 2023). This explained why benefit finding could affect the meaning in life.

### 5.3 Anticipatory grief mediated the relationship between benefit finding and meaning in life

Hypotheses 3, 6 and 7 were supported as the finding indicated a negative effect of anticipatory grief on meaning in life, a negative effect of benefit finding on anticipatory grief, and anticipatory grief mediated the relationship between benefit finding and meaning in life. The link between benefit finding and meaning in life can be elucidated by several factors. Benefit finding prompts patients to reevaluate challenging aspects of their illness positively, embrace role modifications and cultivate an openness to experiencing positive emotions. This proactive approach inhibits the escalation of negative emotional responses, thereby mitigating the effects of anticipatory grief on mental well‐being (Lin et al., 2021). In the medical process, this will provide experience and evidence for psychological interventions to alleviate anticipatory grief and promote the physical recovery of cancer patients. Patients diagnosed with advanced lung cancer face a challenging prognosis characterized by a high likelihood of recurrence and persistent health threats like dyspnea. These factors exacerbate the experience of anticipatory grief, amplifying its impact on patients' emotional well‐being (Cui et al., 2023). Anticipatory grief contributes to a gradual decline in patients' physical condition, serving as an early indicator of illness progression. This heightened apprehension about disease advancement accelerates the onset and evolution of cancer. Consequently, patients experience diminished expectations regarding life expectancy, leading to considerable suffering and loss for both patients and their families. Therefore, patients felt that life had no meaning (Patinadan et al., 2022). In summary, patients exhibiting high levels of benefit finding demonstrate enhanced emotion regulation abilities and are inclined to experience lower levels of anticipatory grief. They exhibit optimism and recognize positive changes resulting from their illness, thereby attributing meaning and value to their lives. This underscores the importance for nursing staff to focus on assessing the benefit‐finding levels of patients with advanced lung cancer during daily care routines. By guiding patients' cognition, nurses can help alleviate anticipatory grief, foster adaptive behaviours towards cancer, encourage the pursuit of life goals and bolster their sense of life's meaning.

### 5.4 Spiritual well‐being mediated the relationship between benefit finding and the meaning in life

Hypotheses 4, 2, and 5 were supported as the finding indicated that benefit finding had a positive effect on spiritual well‐being, spiritual well‐being had a positive effect on meaning in life, and spiritual well‐being mediated the relationship between benefit finding and meaning in life. Previous studies have also confirmed the positive association between benefit finding and spiritual well‐being (Foster et al., 2013; Rong et al., 2022). Steffen found in a study of menopausal women that benefit finding could alleviate physical and psychological symptoms in menopausal women and thus improved spiritual well‐being (Steffen & Soto, 2011). Benefit findings, characterized by positive, intentional motivations and behaviours, could facilitate patients' perception of adapting to adversity, enabling them to view illness as an opportunity for growth. This perspective may initiate a sequence of positive changes aimed at attaining inner comfort and overall well‐being. Consequently, it enhances spiritual well‐being and fosters a state of internal stability (Lassmann et al., 2021). Some studies have shown that spiritual well‐being could effectively improve individual's level of meaning in life (Lovell et al., 2021; Yilmaz, 2019). Spiritual well‐being emphasizes ‘the process of meaning‐making in life’, with its most significant contribution to mental health being the drive for patients to find meaning in their lives. This pursuit offers balance, support and comfort during critical times, enabling individuals to overcome physical and psychological suffering and enhancing their self‐perceived value in life (Connolly & Timmins, 2021). As a result, benefit finding can reassign a positive value to the disease to promote spiritual well‐being. Spiritual well‐being can effectively buffer the adverse consequences of disease stress on patients, maintaining a good psychological state and enabling them to perceive a higher level of meaning in life. In their future clinical work, nursing staff should focus on spiritual well‐being and benefit finding, combining various methods to develop significant life interventions suitable for advanced lung cancer patients, helping them rediscover the meaning in life.

### 5.5 Anticipatory grief and spiritual well‐being had a chain mediating effect between benefit finding and meaning in life

Hypotheses 8 and 9 were supported as the finding indicated that spiritual well‐being negatively affected anticipatory grief and that spiritual well‐being and anticipatory grief had a chain mediating effect between benefit finding and meaning in life. The explanation for the link between spiritual well‐being and anticipatory grief was that a cancer diagnosis was a traumatic stressor that could trigger adverse outcomes. Spiritual well‐being was an essential strength for cancer patients, aiding in their adaptation to the cancer state, fostering a sense of purpose and self‐efficacy (Albusoul et al., 2022; Christian et al., 2019). It also facilitated the creation of a favourable psychological space, assisting patients in overcoming emotional distress and relieving stress associated with physical illness. Consequently, it reduced the level of anticipatory grief (Hiratsuka et al., 2023). According to stress and coping theory (Lazarus, 1993), individuals initially engage in a comprehensive cognitive evaluation of a given stressful situation. Subsequent mental evaluations influence their coping strategies and the outcomes of both physical and psychological adjustment (Lazarus, 1993). In summary, benefit finding can promote a shift in patients' irrational cognitive patterns regarding stressful events and foster an optimistic interpretive style, enhancing spiritual well‐being. Spiritual well‐being reduces anticipatory grief and mitigates the patient's psychological fear of death, ultimately enhancing the patient's sense of life's meaningfulness (Pokpalagon et al., 2022). The implication is that nursing staff should attentively monitor each patient's physical and mental condition, gradually increasing the level of benefit finding to enhance spiritual well‐being. This serves to buffer the adverse effects of anticipatory grief and raise patients' awareness of the meaning of life.

In summary, nurses can implement a series of intervention measures to gradually enhance the sense of meaning in life among advanced lung cancer patients. Initially, they can use cognitive‐behavioural therapy to help individuals identify and change negative thought patterns, fostering a more positive mindset and increasing the level of benefit finding. In the second step, nurses should conduct detailed assessments of the patients' spiritual well‐being to understand their spiritual beliefs, values and needs. Based on the assessment results, nurses can provide spiritual support, including encouraging patients to engage in positive spiritual practices and providing spiritual literature and resources. In the third step, nurses should facilitate the expression of anticipatory grief by patients, encouraging them to articulate their fears and emotions about the end of life. Through dialogue with patients, nurses can assist them in addressing potential anticipatory grief and provide emotional support. In the fourth step, nurses should offer professional counselling services to help patients cope with psychological challenges related to benefit finding, anticipatory grief and spiritual well‐being. Nurses can discuss with patients their views on the meaning and goals of life, assisting them in finding meaningful pursuits at the end of life. This may involve setting small goals, fulfilling wishes or participating in meaningful activities.

## 6 IMPLICATION

Despite the study had limitations, there were implications for caring for patients with advanced lung cancer. Our findings suggested that benefit finding was positively associated with meaning in life. Therefore, the nursing personnel can take targeted measures to enhance the level of benefit finding in patients and thus improve the sense of meaningfulness of life in patients with advanced lung cancer. The study also confirmed multiple mediating pathways, suggesting that nursing personnel should prioritize cultivating benefit finding. They can achieve this by adopting effective interventions to change the mindset of patients with advanced lung cancer. Subsequently, guiding these patients to discover positive meaning in their traumatic experiences through various means becomes essential. Improving the spiritual well‐being of patients with advanced lung cancer, and thereby reducing anticipatory grief, is a crucial process to motivate them to find meaning in their lives. The study's results may offer theoretical guidance for subsequent healthcare professionals to develop interventions for the meaning of life in advanced cancer patients from social and psychological perspectives.

## 7 LIMITATION

First, in this study, only patients with advanced lung cancer in a hospital were selected for investigation, and the sample representativeness was limited. Second, patients completed the questionnaire as a self‐report, which was somewhat subjective and could impact the results. In the future, more objective indicators should be included for analysis. Third, this study was a cross‐sectional study. It may not be able to show the changes in benefit finding, spiritual well‐being, anticipatory grief and meaning in life over time in patients with advanced lung cancer, and longitudinal studies can be conducted in the future for further exploration.

## 8 CONCLUSION

This study elucidated the mechanisms by which spiritual well‐being and anticipatory grief had a chain mediating effect between benefit finding and meaning in the life of patients with advanced lung cancer, providing a new perspective and theoretical basis for research on meaning in the life of interventions related to patients with advanced lung cancer. Therefore, subsequent nursing personnel should focus on these aspects in their clinical work to improve the sense of life meaning in patients with advanced lung cancer.

## AUTHOR CONTRIBUTIONS

Data collection: Qingyue Luo, Fanglin Liu.Data Analysis and Interpretation: Qingyue Luo, Zhaoyu Jiang. Article Drafting: Qingyue Luo. Critical revision of the article: Qingyue Luo, Lan Zhang.

## FUNDING INFORMATION

No additional funds.

## CONFLICT OF INTEREST STATEMENT

The author state that the study was conducted without any business or financial relationship that could be interpreted as a potential conflict of interest.

## ETHICAL APPROVAL

The First Affiliated Hospital of Jinzhou Medical University's Ethics Committee approved this study. Ethics approval number is JZMULL2023050 and written informed consent was obtained from each patient before participation in this study. There were no details of ethical approval for research with human subjects.

## Data Availability

The data in this article were checked by the authors to ensure compliance with journal statistical guidelines. Due to privacy guidelines, the data for this study was not publicly available, but it can be obtained from the author for reasons.
